# The long non-coding RNA Linc-GALH promotes hepatocellular carcinoma metastasis via epigenetically regulating Gankyrin

**DOI:** 10.1038/s41419-019-1348-0

**Published:** 2019-01-28

**Authors:** Xiaoliang Xu, Yun Lou, Junwei Tang, Yue Teng, Zechuan Zhang, Yin Yin, Han Zhuo, Zhongming Tan

**Affiliations:** 1 Medical School of Southeast University, Nanjing, Jiangsu P.R. China; 2 Department of Hepatobiliary Surgery, The Affiliated Drum Tower Hospital of Nanjing University Medical School, Nanjing, 210093 Jiangsu Province P.R. China; 3 Key Laboratory of Living Donor Liver Transplantation, Department of Liver Surgery, National Health and Family Planning Commission, The First Affiliated Hospital of Nanjing Medical University, Nanjing, Jiangsu P.R. China; 4 Department of Medical Oncology, Jiangsu Cancer Hospital & Jiangsu Institute of Cancer Research & Nanjing Medical University Affiliated Cancer Hospital, Nanjing, 210009 Jiangsu P.R. China

## Abstract

Hepatocellular carcinoma (HCC) is the most prevalent subtype of liver cancer, and it is characterized by high rate of metastasis and recurrence. Recent studies have boosted our understanding that Gankyrin contributes to both of these pathological properties, but the mechanisms underlying its aberrant regulation are poorly understood. Recently, many long noncoding RNAs (lncRNAs) have been reported to be involved in regulating the expression of oncogenes and anti-oncogenes through various mechanisms. Here, using transcriptome microarray analysis, we identified a long intergenic noncoding RNA termed Linc-GALH that was highly expressed and concordance with Gankyrin expression in HCC. In addition, we revealed that Linc-GALH was an independent unfavorable prognostic indicator for HCC, followed functional experiments showed that Linc-GALH promoted HCC cells migration and invasion in vitro, and enhanced lung metastasis ability of HCC cells in vivo. Mechanistically, we found that Linc-GALH could regulate the expression of Gankyrin through controlling the methylation status of Gankyrin by adjusting the ubiquitination status of DNMT1 in HCC. Collectively, our results demonstrated the role and functional mechanism of Linc-GALH in HCC, and indicated that Linc-GALH may act as a prognostic biomarker and potential therapeutic target for HCC.

## Introduction

Hepatocellular carcinoma (HCC) is emerging as the fifth most common carcinoma and the third leading cause of cancer-associated mortality worldwide^1^. Despite advances in early diagnosis and therapeutic treatments for HCC, the early diagnosis rate and long-term survival rate remains poor^2^. Aggressiveness, invasiveness (in particular, intrahepatic) and frequent postoperative recurrence are the most significant characteristics of HCC^3^. Based on this, elucidation of the mechanisms underlying HCC initiation, progression and metastasis is benefit to improve early diagnosis and test prognosis.

Gankyrin (standard nomenclature is PSMD10), a small protein with seven ankyrin-repeat domains, was originally identified as a regulatory subunit of the 26 S proteasome complex^4^. Gankyrin was illustrated to activate Akt through regulating RhoA/ROCK signaling pathway and then promote tumorigenesis and metastasis of HCC^5^. In addition, Gankyrin could activate PI-3K/Akt/HIF1α pathway to promote the expression of Twist1, VEGF and MMP2, thus accelerated the EMT transformation of hepatoma cells^6^. It has also been found high expressed in other malignant tumors such as lung cancer, breast cancer, colon cancer and so on^7–10^. A study published in 2014 revealed that the expression of Gankyrin in patients with metastatic gastric cancer was significantly lower than that in patients without metastatic gastric cancer. Furthermore, high levels of methylation of Gankyrin were found in metastatic gastric cancer tissues, which were significantly higher than those in non-metastatic gastric cancer patients and controls^11^. Studies have shown that aberrant DNA methylation alteration always participates in the abnormal low expression of tumor suppressor genes in HCC^12–14^. However, at present, the methylation regulation of Gankyrin in HCC and whether it is involved in the regulation of metastasis of HCC has not been reported.

Long noncoding RNAs (IncRNAs) are a class of transcripts with at least 200nt in length, which have no or limited protein coding ability^15^. Many lncRNAs have been proved to play important roles in controlling the expression of oncogenes and anti-oncogenes and then participate in the tumorigenesis and development of various tumors, including HCC^16–19^. Recent studies have shown that the interaction between lncRNAs and DNA methylation plays an important role in tumor biology^20–22^.

In the present study, we attempt to investigate whether DNA methylation can regulate the expression of Gankyrin in hepatocellular carcinoma and whether there are lncRNAs which might be involved in this regulation. We showed that a lincRNA (ENST00000413791.1) was concordance with Gankyrin expression in HCC, and named it as Gankyrin Associated LincRNA in Hepatocellular carcinoma (Linc-GALH). We also explored the function of Linc-GALH by using in vitro and in vivo assays, and further investigated thoroughly the potential regulatory mechanism between Gankyrin and Linc-GALH. Eventually we found that Linc-GALH could regulate the expression of Gankyrin through controlling the methylation status of Gankyrin by adjusting the ubiquitination status of DNMT1 in HCC.

## Results

### Linc-GALH expression accordance with Gankyrin is upregulated in HCC tumor tissues

**Fig. 1:**
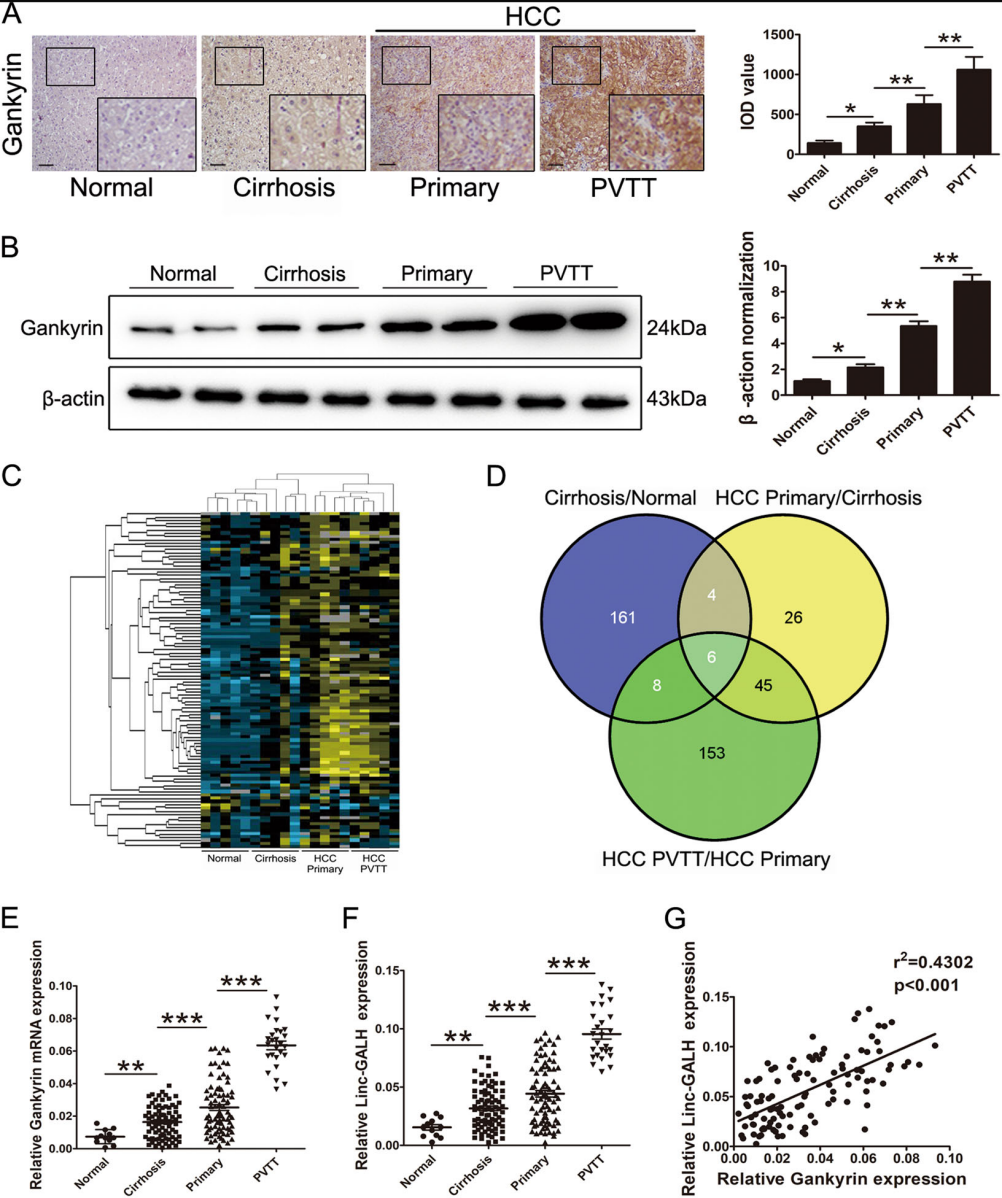
Linc-GALH expression accordance with Gankyrin is upregulated in HCC tumor tissues. **a** Paraffin sections from 108 HCC tissues (82 primary HCC patients; 26 HCC patients with PVTT, PVTT, portal vein tumor thrombus), liver cirrhosis tissues (*n* = 81) and 12 normal liver tissues were used to detect Gankyrin expression via IHC with IOD value examined based on IHC (original magnification × 200). **b** Gankyrin protein expression in normal liver tissues, liver cirrhosis tissues, primary HCC tumor and HCC with PTVV tissues was analyzed by western blot with IOD valve. **c** Hierarchical clustering analysis was used to identify the differential expression of lncRNAs in the 4 groups (normal liver tissues, liver cirrhosis tissues, primary liver cancer tissues and liver cancer with PVTT tissues, each group include 5 samples) microarray data (greater than two-fold). **d** VENNY analysis identified 6 lncRNAs which were all aberrant upregulated in the three comparisons (cirrhosis liver tissues compared with normal liver tissues; primary HCC tissues compared with cirrhosis liver tissues; tissues from HCC patients with PVTT compared with primary HCC tissues). **e** Relative expression of Gankyrin mRNA in the above tissues. **f** Relative expression of Linc-GALH was detected in the above tissues. **g** A positive correlation between expression levels of Linc-GALH and Gankyrin in HCC tissues was determined by Person analysis (*n* = 108, *r*^2^ = 0.4302, *P* < 0.001). *** *P* < 0.001; **P < 0.01 and *P < 0.05. *P* < 0.05 was regarded as statistically significant

Previous studies revealed that Gankyrin was an oncogene in HCC^6^, to reconfirm this, we detected the expression level of Gankyrin in normal liver tissues (*n* = 12), liver cirrhosis tissues (*n* = 81), primary liver cancerous tissues (*n* = 82) and hepatocellular carcinoma with portal vein tumor thrombus (PVTT) tissues (*n* = 26) by using immunohistochemical assays, western blot and qRT-PCR (Figs. 1a, b, e). To explore lncRNAs that might contribute to the aberrant expression of Gankyrin in HCC, tissues from four groups (normal liver tissues, liver cirrhosis tissues, primary liver cancer tissues and liver cancer with PVTT tissues, each group include five samples) were collected and then assessed by using lncRNA microarrays. As shown in Fig. 1c, hierarchical clustering analysis showed the differential expression of lncRNAs in the 4 groups from microarray data (greater than two-fold). Filtered by *P*-value and fold change (*P* < 0.05 and fold change >3), we identified 179 lncRNAs aberrant upregulated in cirrhosis liver tissues compared with normal liver tissues, 81 lncRNAs aberrant upregulated in primary HCC tissues compared with cirrhosis liver tissues and 212 lncRNAs aberrant upregulated in tissues from HCC patients with PVTT compared with primary HCC tissues. As shown in Fig. 1d, followed VENNY analysis identified 6 lncRNAs which were all aberrant upregulated in the three comparisons mentioned above (The detailed information of these 6 lncRNAs were listed in supplementary table 3). Next, to further explore the expression levels of the 6 lncRNAs in different liver tissues, we expanded the sample size in each group (10 normal liver tissues, 10 cirrhosis liver tissue, 10 primary liver cancer tissues and 10 tissues from HCC patients with PVTT) and conducted qRT-PCR assays (Supplementary Figure 1). As presented in Supplementary figure 2, followed Pearson correlation analysis revealed that the expression of ENST00000413791.1 showed the greatest consistence with the expression of Gankyrin in HCC tissues, so we named it as Gankyrin Associated LincRNA in Hepatocellular carcinoma (Linc-GALH). As presented in Fig. 1f, to further confirm the aberrant expression of Linc-GALH, we expanded sample size and examined the expression level of Linc-GALH in each group mentioned above by using qRT-PCR, and the followed Pearson correlation analysis further verified the positive correlation relationship between the expression of Linc-GALH and Gankyrin in HCC tissues (Fig. 1g).

### Linc-GALH expression is correlated with the prognosis of patients with HCC

**Fig. 2:**
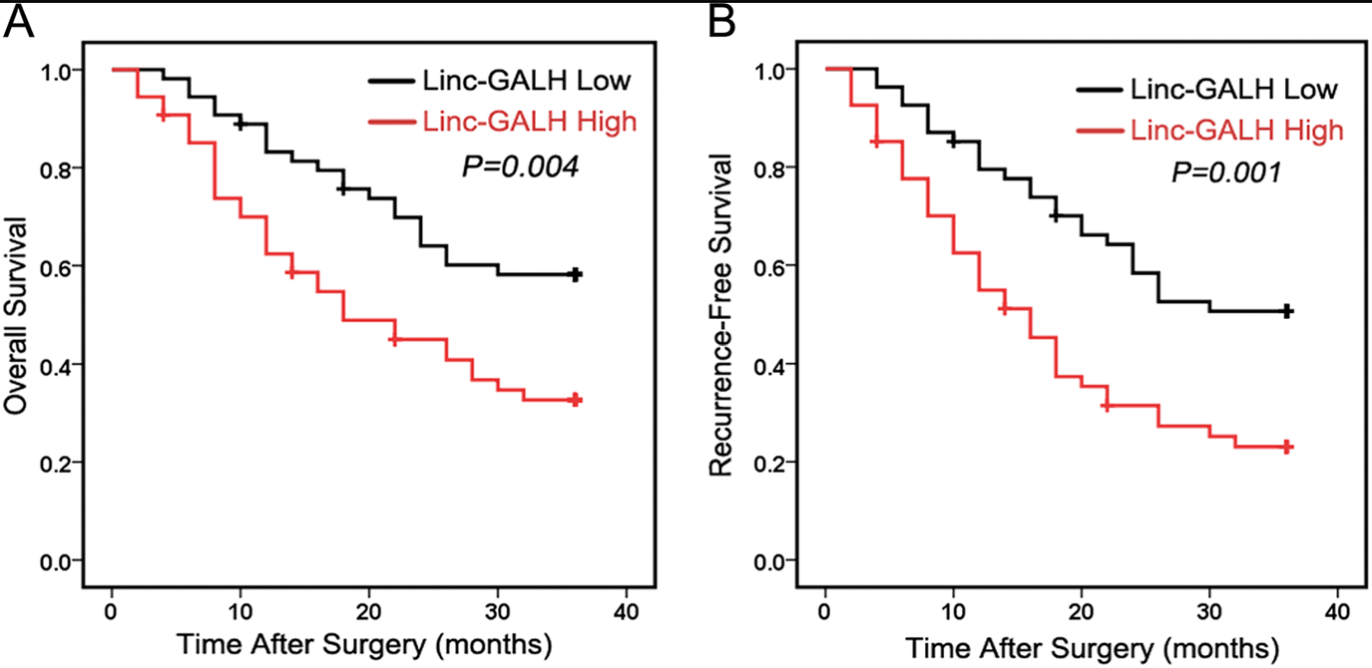
Linc-GALH expression is correlated with the prognosis of patients. **a**, **b** The overall survival and recurrence-free survival rates of 108 HCC patients were compared between low-Linc-GALH and high-Linc-GALH groups

To detect the association between Linc-GALH expression and the clinical characteristics of HCC patients, we divided all patients into Linc-GALH High group and Linc-GALH Low group according to the median value of Linc-GALH expression in tumor tissues of 108 HCC patients and the results were summarized in Table 1. We found that Linc-GALH expression was significantly correlated with intrahepatic metastasis (*P* = 0.040), vascular invasion (*P* = 0.012) and distant metastasis ability (*P* = 0.021). Correlation analysis of Linc-GALH with clinicopathological characteristics indicated that Linc-GALH was highly correlated with HCC metastasis. In addition, Kaplan–Meier analysis was used to detect the influence of Linc-GALH on the survival of HCC patients, our findings revealed that patients with high Linc-GALH expression had poorer prognosis than those with low Linc-GALH expression with shorter overall (*P* = 0.004) and recurrence-free (*P* = 0.001) survival (Fig. 2a, b).

**Table 1:** Relationship between intratumoral Linc-GALH expression and clinicopathologic features

Characteristics	Linc-GALH expression		*P* value
	Low(*N* = 54)	High(*N* = 54)	
Age(years)			0.564
<50	25	28	
≥50	29	26	
Gender			0.588
Male	47	45	
Female	7	9	
AFP(ng/ml)			0.380
≤20.0	16	12	
> 20.0	38	42	
Tumor size(cm)			0.121
≤5	28	20	
> 5	26	34	
HbsAg			0.375
Negative	8	5	
Positive	46	49	
Anti-HCV			0.647
Negative	51	52	
Positive	3	2	
Liver cirrhosis			0.505
Yes	39	42	
No	15	12	
Vascular invasion			0.012^*^
Yes	20	33	
No	34	21	
Intrahepatic metastasis			0.040^*^
Yes	8	17	
No	46	37	
Distant metastasis			0.021^*^
Yes	2	9	
No	52	45	
Edmondson			0.186
I-II	17	11	
III-IV	37	43	

The median expression level of Linc-GALH was used as the cutoff

*Indicates *P* value < 0.05

### Linc-GALH promotes the migration and invasion abilities of HCC cells in vitro

**Fig. 3:**
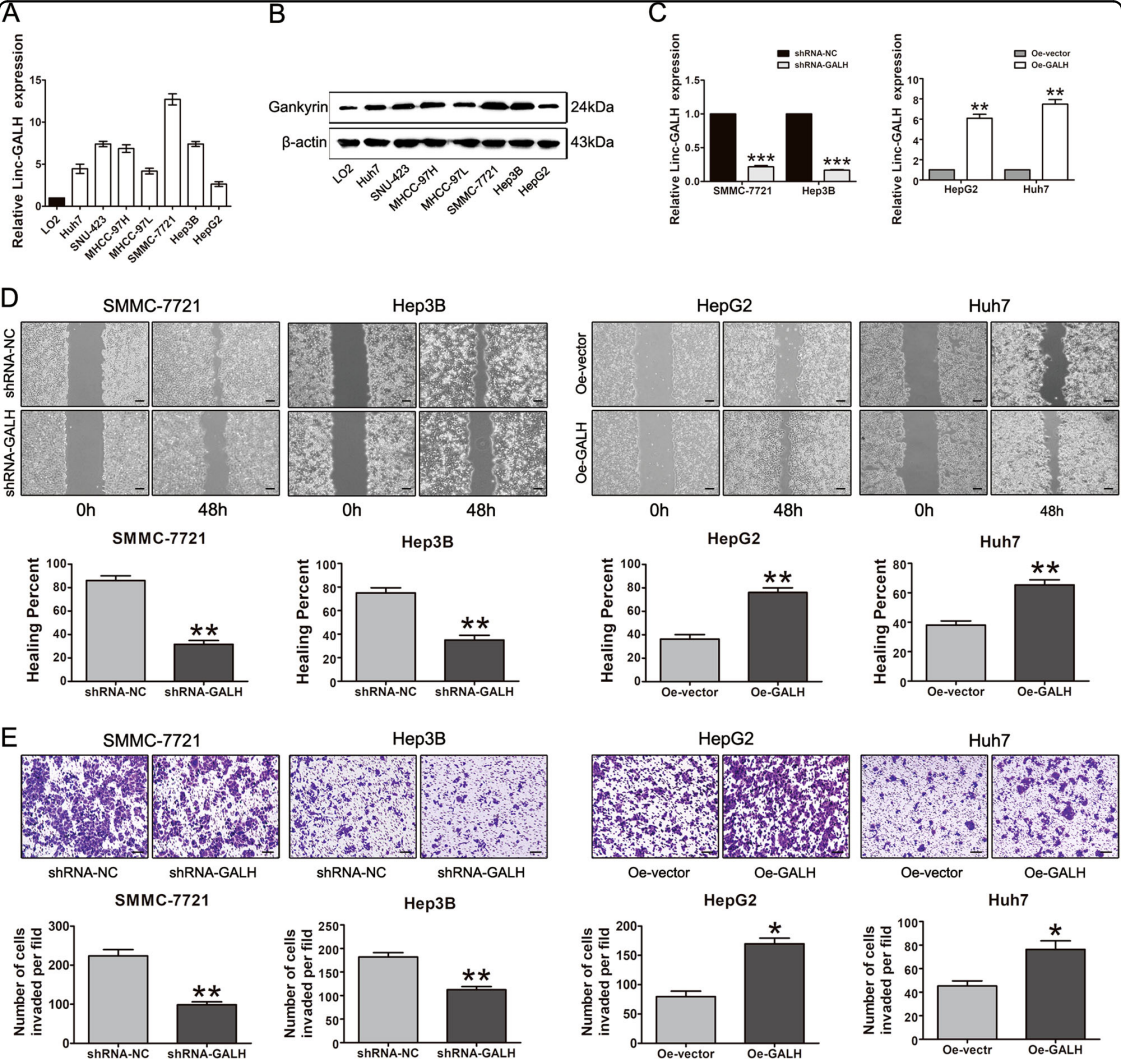
Linc-GALH promotes the migration and invasion abilities of HCC cells in vitro. **a** Relative expression of Linc-GALH in human LO2 hepatocytes and HCC cell lines was detected by qPCR. **b** The protein level of Gankyrin in human LO2 hepatocytes and HCC cell line was examined by western blot. **c** Gene silencing of Linc-GALH using Linc-GALH shRNA in SMMC-7721 and Hep3B cells and gene ectopic expression of Linc-GALH in HepG2 and Huh7 cells using overexpression lentivirus and the transfection efficiency was detected by qPCR. **d** Wound healing assays was performed to investigate the in vitro migration abilities of SMMC-7721 and Hep3B cells transduced with Linc-GALH shRNA, HepG2 and Huh7 cells transduced with Linc-GALH Lv respectively (original magnification × 40). **e** The in vitro invasion abilities of SMMC-7721, Hep3B, HepG2 and Huh7 cells lentivitally transduced with Linc-GALH shRNA or Lin-GALH Lv were examined using transwell assays (original magnification × 100). Each experiment was performed in triplicate and data from the experimental and control groups compared. *P < 0.05 and **P < 0.01. *P* < 0.05 was regarded as statistically significant

Next, to investigate the effects of Linc-GALH on the biological behaviors of HCC cells, we explored the expression spectrum of Linc-GALH and Gankyrin in HCC cell lines. The RNA level of Linc-GALH in HCC cell lines was significantly increased in all seven HCC cell lines (Fig. 3a). Also, we examined the protein level of Gankyrin in HCC cell lines and normal liver cell line LO2 (Fig. 3b). Thus, we selected SMMC-7721 and Hep3B cell lines to construct the Linc-GALH knockdown model, HepG2 and Huh7 cell lines to construct the Linc-GALH overexpression model separately. The transduction efficiencies were measured by qPCR, compared with normal control (shRNA-NC), shRNA-GALH reduced the expression of Linc-GALH by as much as 20% in SMMC-7721 and Hep3B cell lines, Oe-GALH increased the expression of Linc-GALH about 6 times compared with Oe-vector in HepG2 cell line and 7 times compared with Oe-vector in Huh7 cell line (Fig. 3c and Supplementary Figure 3A).

Correlation analysis of Linc-GALH with clinic pathological characteristics indicated that Linc-GALH might promote HCC metastasis. To investigate whether Linc-GALH participated in the regulation of the migration and invasion abilities of HCC cells, Linc-GALH stable knockdown and upregulation HCC cells constructed above were subjected to wound healing and invasion assays. Obviously, the wound closure ability was significantly inhibited when Linc-GALH was knocked down in SMMC-7721 and Hep3B cells, whereas cells in Linc-GALH overexpressed HepG2 and Huh7 cells migrated faster than those in the control group (Fig. 3d). We also found that transfection with shRNA-GALH suppressed the invasion of SMMC-7721 and Hep3B cells and upregulation of Linc-GALH significantly enhanced the invasion of HepG2 and Huh7 cells in the transwell assays (Fig. 3e). Our results clearly supported a promoting effect of Linc-GALH on migration and invasion in HCC cells in vitro.

### Linc-GALH enhances the lung metastasis ability of HCC cells in vivo

**Fig. 4:**
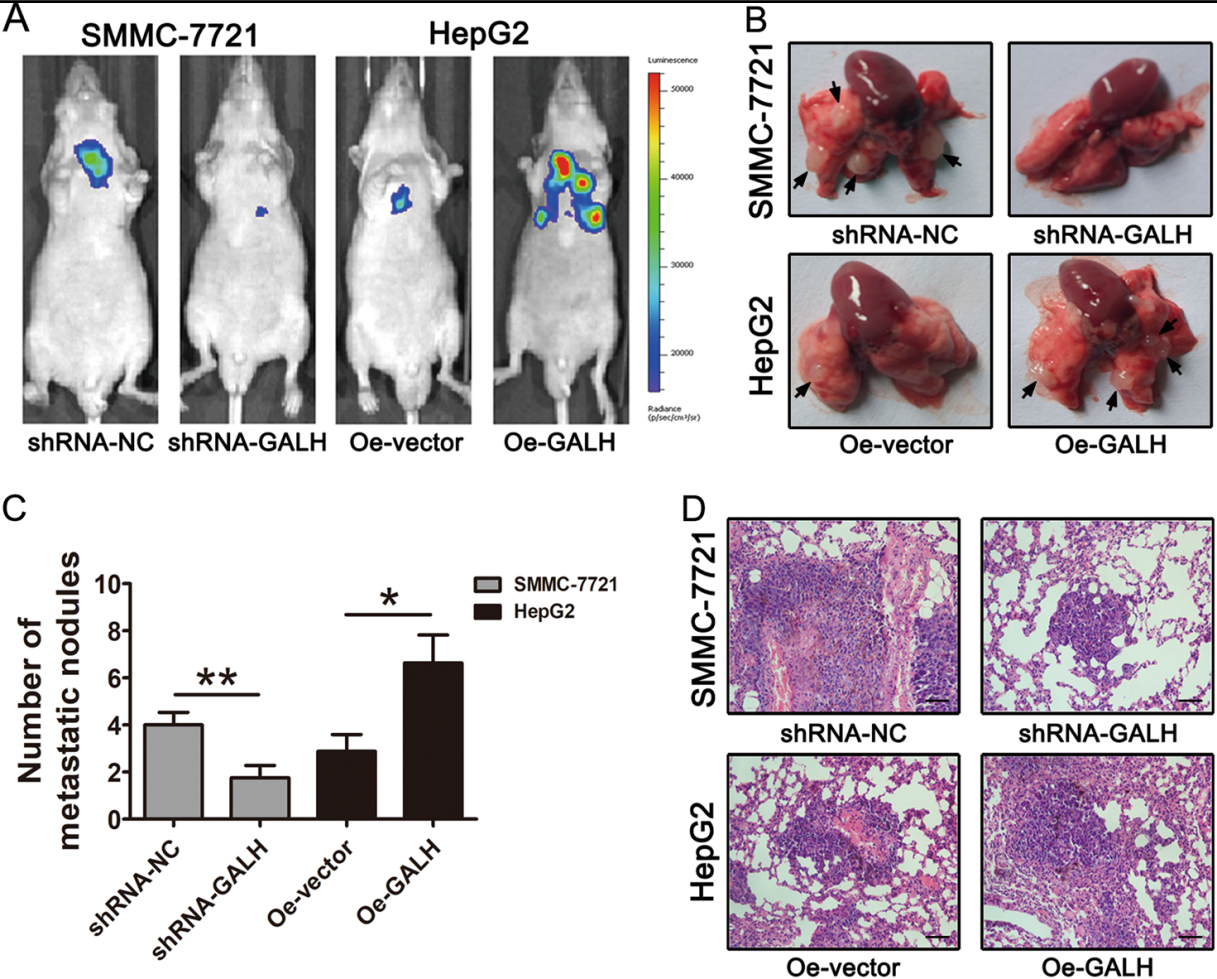
Linc-GALH enhances the lung metastasis ability of HCC cells in vivo. **a** A lung metastasis model was established in which mice (8 in each group) were injected with HCC cells (5 × 10^6^ cells suspended in 200 μL PBS) through the tail vein and the lung metastasis was investigated respectively using the IVIS Lumina II system. Representative images of a mouse in each group were presented. **b** Photomicrographs were taken for lung metastasis in nude mice. Representative images of a mouse in each group were presented. Arrowheads indicate the metastatic nodes. **c** Metastatic tumors with volumes >2 mm^3^ were identified and compared in each group. **d** All the results of lung colonization were validated by the histological examination (H&E) (original magnification × 200). Experiments were performed in triplicate independently, **P < 0.01; *P < 0.05. *P* < 0.05 was regarded as statistically significant

To further explore the function of Linc-GALH in vivo, Linc-GALH knockdown and controlled SMMC-7721 cells and Linc-GALH overexpressed and controlled HepG2 cells labeled with luciferase were injected through the tail vein of mice to establish a lung metastasis model. Compared with the group produced from SMMC-7721-shRNA-NC cells, the number of lung metastatic nodules in mice decreased dramatically in the SMMC-7721-shRNA-GALH group. Moreover, Linc-GALH overexpression significantly enhanced the pulmonic metastases of HCC HepG2 cells (Figs. 4a-c). The same trend was also achieved by histopathological analysis by H&E staining (Fig. 4d). Taken together, our results revealed that Linc-GALH enhanced the lung metastasis ability of HCC cells in vivo.

### Linc-GALH regulates the Gankyrin gene methylation level in both HCC tissues and cells

A number of oncogenes and anti-oncogenes have been reported to be regulated by DNA methylation in various cancers^23–25^. High levels of methylation of Gankyrin were found in metastatic gastric cancer tissues, which were significantly higher than those in non-metastatic gastric cancer patients and controls^11^. In addition, many lncRNAs were reported to regulate gene expression through adjusting DNA methylation status of target genes^20,24,26^. Since the expression of Linc-GALH was accordance with Gankyrin in HCC patients, we got a hypothesis that DNA methylation might participate in the expression of Gankyrin in HCC patients and Linc-GALH might be involved in this regulation.

**Fig. 5:**
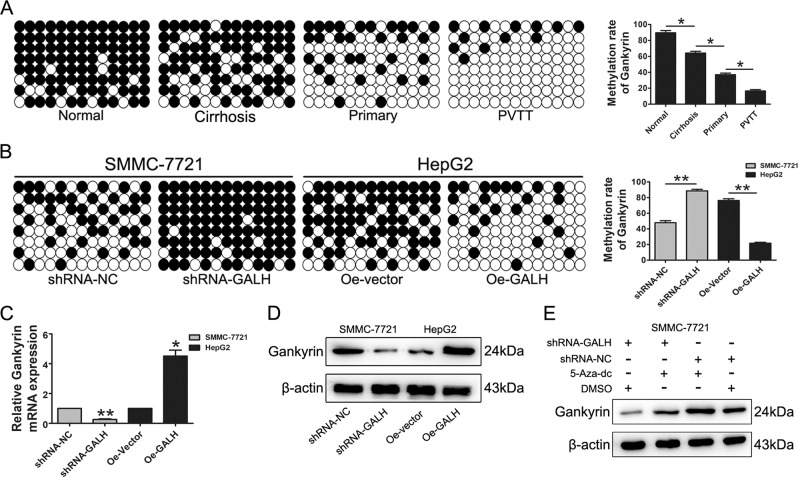
Linc-GALH regulates the Gankyrin gene methylation level in both HCC tissues and cells. **a** The bisulfite sequencing method was applied to examine the methylation rate of the Gankyrin promoter in normal liver tissues, cirrhosis liver tissues, primary HCC tumor tissues and the tumor tissues of HCC patients with PVTT. Representative results of each group were shown and the difference of each group was analyzed. **b** Representative bisulfite sequencing analysis of the Gankyrin promoter in HCC cells transduced with Linc-GALH shRNA or Linc-GALH Lv, respectively. **c**, **d** Relative mRNA and protein levels of Gankyrin in HCC cells upon Linc-GALH alteration were detected by qPCR and western blot assays, respectively. **e** The protein level of Gankyrin was evaluated in SMMC-7721 cells treated with 5-aza-dC (DAC) and DMSO as control. Each experiment was performed in triplicate independently; **P < 0.01. *P* < 0.05 was regarded as statistically significant

To validate the hypothesis, firstly, we investigated the methylation regulatory potential in the promoter region of Gankyrin based on CpG island prediction using MethPrimer (http://www.urogene.org/methprimer/) and USCS genome bioinformatics (http://genome.ucsu.edu) (Supplementary Figure 3B). The methylation primer was presented in Supplementary table 1. And then, normal liver tissues (*n* = 10), primary HCC tumor tissues (*n* = 20), cirrhosis liver tissues (*n* = 20) and tumor tissues of HCC patients with portal vein tumor thrombus (*n* = 20) were included in the bisulfate PCR. As shown in Fig. 5a, the methylation rate of Gankyrin promoter was significantly lower in HCC tumor tissues compared with the cirrhosis liver tissues and normal liver tissues and further decreased in the tumor tissues of HCC patients with portal vein tumor thrombus. Next, we further conducted the bisulfate sequencing to analysis the CpG methylation in the promoter of Gankyrin in Linc-GALH down-regulated and up-regulated HCC cells. Our results revealed that SMMC-7721 cells transduced with Linc-GALH shRNA showed more intensively hypermethylated CpG islands in the promoter of Gankyrin, and Gankyrin CpG islands were more hypomethylated in Linc-GALH over-expressed HepG2 cells compared with the control group (Fig. 5b), suggesting that Linc-GALH was involved in regulating Gankyrin gene methylation. Since DNA methylation can regulate gene expression at transcriptional level, we then performed qPCR and western blot assays to detect the Gankyrin expression on transcriptional and translational level upon Linc-GALH alteration in HCC cells. We found that overexpression of Linc-GALH evidently upregulated Gankyrin expression in HepG2 cells, whereas knock down of Linc-GALH remarkably suppressed Gankyrin expression in SMMC-7721 cells on transcriptional and translational levels (Fig. 5c-d).

Numerous studies have proved that DNA methyltransferases (DNMTs, including DNMT1, DNMT3A and DNMT3B) are the main factors involving in the regulation of oncogenes and anti-oncogenes in various tumors including HCC. 5-aza-2′-deoxycytidine (5-Aza-dC), an inhibitor of DNMTs which can bind covalently to the DNMTs and irreversibly inhibit their function has been used extensively in clinical trials for cancer treatment. To validate whether DNMTs participate in the regulatory relationship between Linc-GALH and Gankyrin, 5-Aza-dC was used to treat HCC cells. We found that treatment of HCC cells with 5-Aza-dC led to the restoration of Gankyrin expression in both SMMC-7721 Linc-GALH knockdown cells and the control cells (Fig. 5e), suggesting DNMTs was involved in the regulatory relationship between Linc-GALH and Gankyrin. Taken together, our data indicated that Linc-GALH could regulate Gankyrin expression through modulating DNA methylation and DNMTs might take part in this mechanism.

### Linc-GALH induces EMT and is mainly mediated by Gankyrin in HCC

**Fig. 6:**
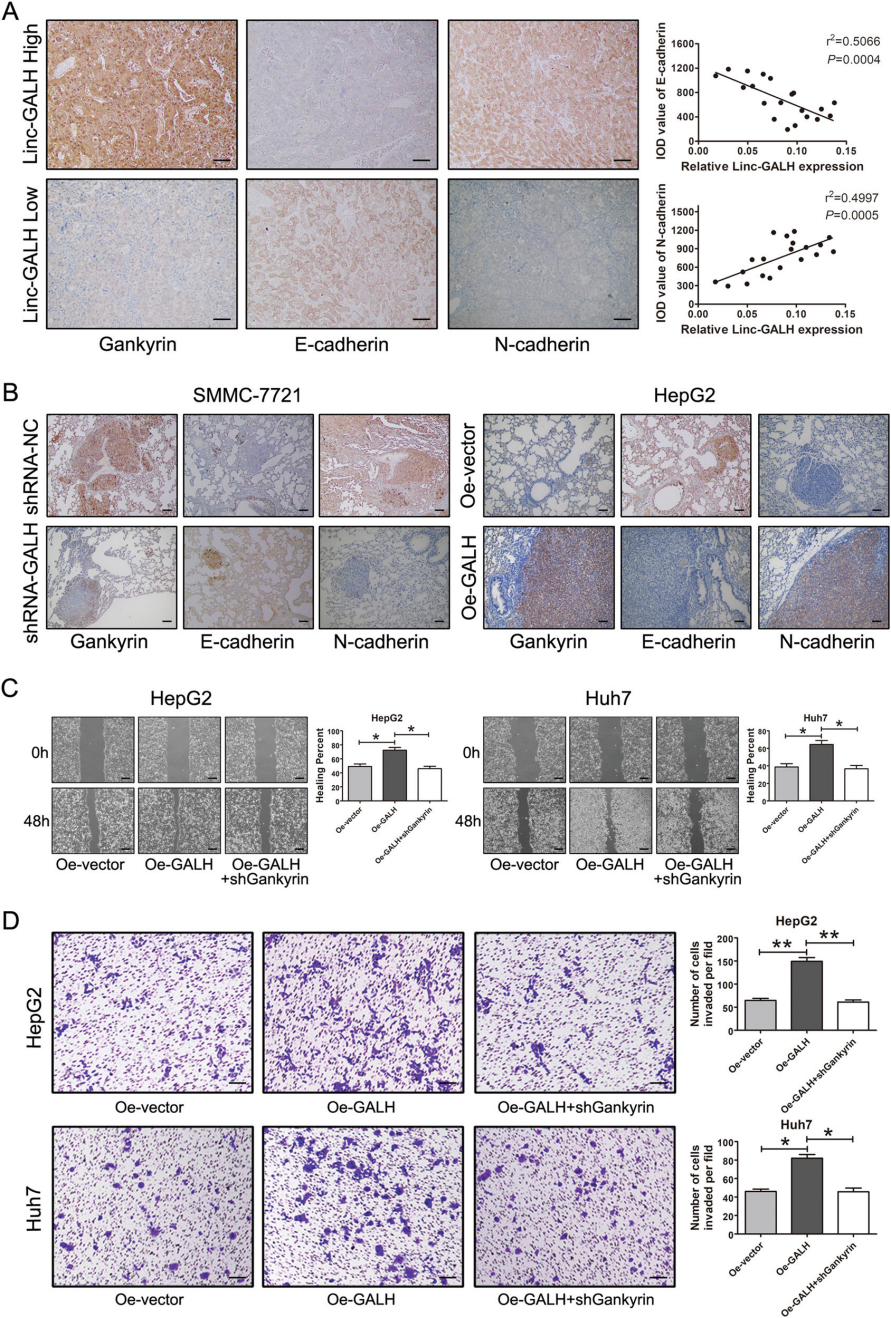
Linc-GALH induces EMT and mainly mediated by Gankyrin in HCC. **a** IHC assessment of intensity of Gankyrin, E-cadherin and N-cadherin in 10 Linc-GALH-High and 10 Linc-GALH Low sections from HCC patients (original magnification × 200). A strong positive correlation of Linc-GALH expression with N-cadherin levels (*r*^2^ = 0.4997, *P* = 0.0005) and a strong negative correlation of Linc-GALH expression with E-cadherin levels (*r*^2^ = 0.5066, *P* = 0.0004) was gained. **b** Representative pictures of immunochemical staining of lung sections for Gankyrin, E-cadherin and N-cadherin in the Linc-GALH knockdown and controlled SMMC-7721 cells and Linc-GALH overexpressed and controlled HepG2 cells (original magnification × 200). **c** Wound healing assays was performed to examine the in vitro migration abilities of Linc-GALH overexpressed HepG2 Huh7 cells transduced with shGankyrin (original magnification × 40). **d** The in vitro invasion abilities of Linc-GALH overexpressed HepG2 and Huh7 cells transduced with shGankyrin were investigated using transwell assays (original magnification × 100). Each experiment was performed in triplicate independently; **P < 0.01. *P* < 0.05 was regarded as statistically significant

**Fig. 7:**
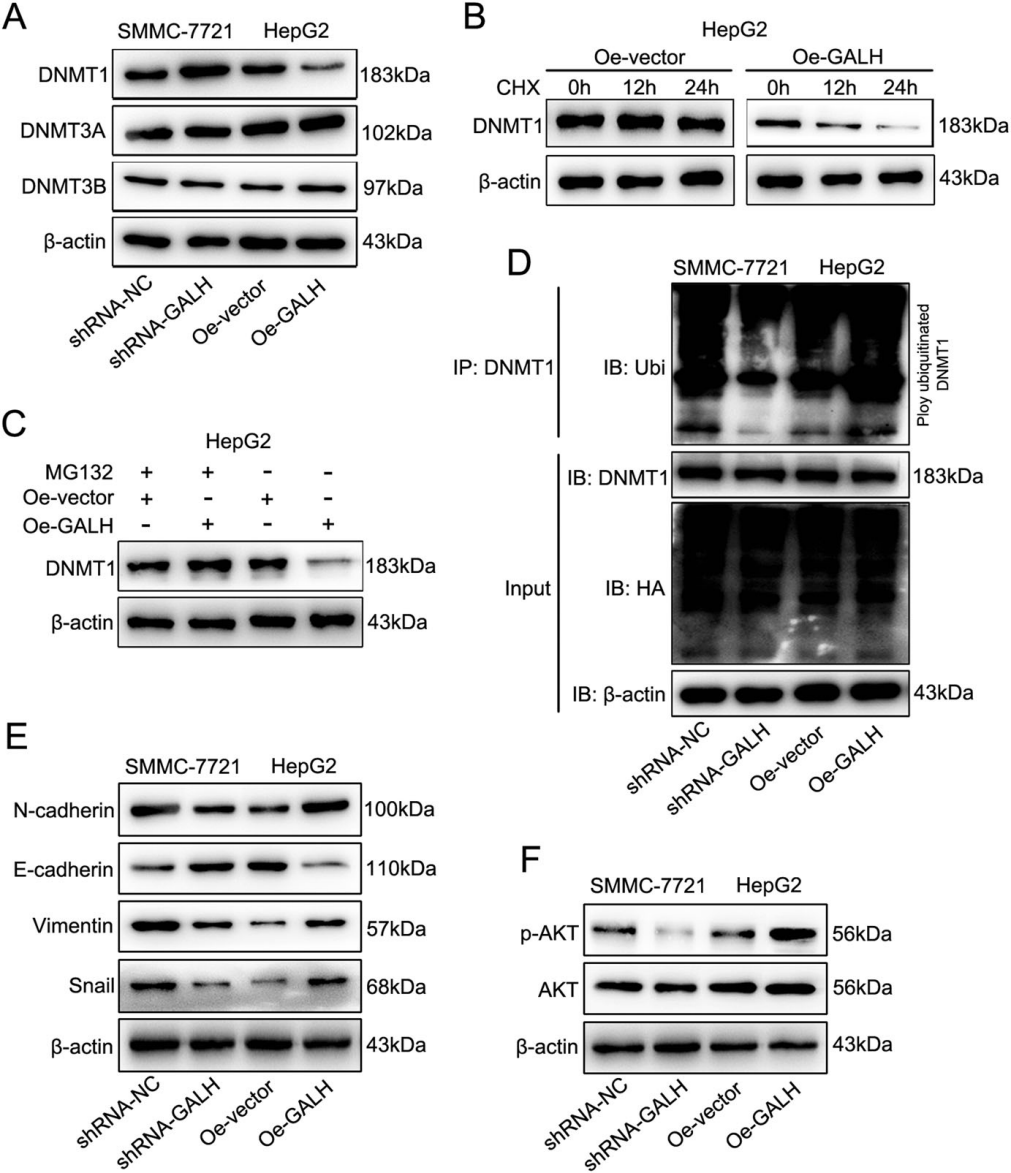
Linc-GALH regulates the ubiquitination of DNMT1 and involves the regulation of AKT signaling. **a** Expression levels of DNMT1, DNMT3A and DNMT3B were detected by western blot in HCC cells when Linc-GALH was knockdown or upregulated. **b** Expression of DNMT1 was confirmed by western blot in HepG2 cells treated with cycloheximide (100 μg/mL). **c** HepG2 cells transfected with Linc-GALH Lv or the control lentivirus were cultured with or without MG132, and western blot was conducted using anti-DNMT1 antibody. **d** The lysates of HCC cells transduced with Linc-GALH shRNA or Linc-GALH Lv lentivirus and their control group were conducted to immunoprecipitated with anti-DNMT1 antibodies to detect polyubiuitination of DNMT1 with anti-Ub antibodies. **e** The expression levels of EMT related genes were examined by western blot in HCC cells upon Linc-GALH alteration. **f** p-AKT was evaluated by immunoblot analysis with AKT protein and β-actin as controls

Epithelial-mesenchymal transition (EMT) has been recognized as a critical regulator of metastasis in HCC. In consideration of that Gankyrin has been reported to enhance EMT transition in HCC, we guess that Linc-GALH might be involved in regulation of EMT. To validate this hypothesis, we randomly selected 20 HCC tissues (10 from Linc-GALH High group and 10 from Linc-GALH Low group) and conducted immunochemical staining for Gankyrin, E-cadherin (an epithelial marker) and N-cadherin (a mesenchymal marker). The followed Pearson correlation analysis revealed a strong positive correlation of Linc-GALH expression with N-cadherin levels (*r*^2^ = 0.4997, *P* = 0.0005) and a strong negative correlation of Linc-GALH expression with E-cadherin levels (*r*^2^ = 0.5066, *P* = 0.0004) (Fig. 6a). Next, we investigated the presence of EMT in vivo by staining the EMT markers in lung sections. There was increased Gankyrin and N-cadherin expression but decreased E-cadherin expression in lung sections with overexpressed Linc-GALH, whereas decreased Gankyrin and N-cadherin expression and increased E-cadherin expression in lung sections with Linc-GALH knockdown (Fig. 6b). In addition, the western blot assays using antibodies of EMT related genes verified that overexpression of Linc-GALH enhanced the expression of the mesenchymal markers N-cadherin, Vimentin and Snail, but reduced the expression of epithelial marker E-cadherin (Fig. 7e). To verify whether the function of Linc-GALH on EMT in HCC cells was mediated by Gankyrin, we knockdown Gankyrin expression in Linc-GALH overexpressed HepG2 and Huh7 cells, and the transfection efficiencies were shown in Supplementary Figure 3C. The followed wound healing and invasion assays revealed that knockdown of Gankyrin restored the function of Linc-GALH overexpression in HepG2 and Huh7 cells (Fig. 6c, d). Collectively, these results indicated that Linc-GALH is capable of regulating EMT phenotype of HCC both in vitro and in vivo and mainly mediated by Gankyrin.

### Linc-GALH regulates the ubiquitination of DNMT1 and involves the regulation of AKT signaling

To determine whether all the DNMTs or one of them play a part in the regulatory relationship between Linc-GALH and Gankyrin, we detected their expression upon Linc-GALH alteration in HCC cells. Interestingly, we found that only the expression of DNMT1 changed with the alteration of Linc-GALH in HCC cells (Fig. 7a), indicating that DNMT1 was the main regulatory factor in the mechanism and Linc-GALH might participate in the regulation of DNMT1. Next, we investigated the effects of Linc-GALH on DNMT1, as shown in Fig. 7a, we found that knockdown of Linc-GALH significantly enhanced DNMT1 protein level compared with shRNA-NC in SMMC-7721 cells, and Linc-GALH overexpression obviously reduced DNMT1 protein level in HepG2 cells. To further validate the influence of Linc-GALH on DNMT1 protein expression, cycloheximide (CHX, a protein biosynthesis inhibitor) was used to treat HepG2 cells, we found that the suppression of DNMT1 protein by Linc-GALH was notably reduced by CHX (Fig. 7b). In addition, HepG2 cells were also treated by MG132 (a widely used proteasome inhibitor) which almost entirely blocked the decrease in DNMT1 protein expression caused by Linc-GALH (Fig. 7c). Based on the results, we summed up a corollary that Linc-GALH reduced DNMT1 expression through a post-transcriptional regulation, such as phosphorylation, acetylation or ubiquitination.

Previous studies have revealed that lncRNAs can regulate the degradation of DNMT1 by controlling its ubiquitination status. Since our results proved that Linc-GALH could regulate the protein level of DNMT1, we speculated that this regulation mechanism might also exist in our study. To verify this hypothesis, we conducted immunoprecipitation assays with anti-DNMT1 antibody in HCC cells after Linc-GALH was knockdown or overexpressed, and the ubiquitination status of DNMT1 was detected by anti-ubiquitin antibody. We found that ubiquitination of DNMT1 was decreased when Linc-GALH was overexpressed and increased when Linc-GALH was knockdown (Fig. 7d), suggesting that Linc-GALH could regulate DNMT1 expression by controlling ubiquitination. Activation of AKT pathway was validated to be responsible for the carcinogenic effect of Gankyrin in HCC, so we analyzed the phosphorylated status of AKT in HCC cells upon Linc-GALH alteration. We found that p-AKT signal was significantly higher in HepG2-Oe -GALH cells than HepG2-Oe-vector cells, whereas knockdown of Linc-GALH remarkably suppressed p-AKT expression in SMMC-7721 cells (Fig. 7f). In conclusion, we found that Linc-GALH regulated the ubiquitination of DNMT1 and then was involved in the regulation of AKT signaling.

## Discussion

HCC is a group of diverse heterogeneous diseases arising through various molecular pathways^3^. This heterogeneity determines tumor prognosis and response to therapy and brings great challenges not only in studying the molecular basis of the disease, but also in clinical patient management. To date, there are few reliable markers available to accurately predict prognosis of HCC. So there is a strong need to identify novel biomarkers that better conduct the option of treatments and predict overall survival of HCC patients.

Gankyrin is an oncogenic protein showing the documented roles in initiation, promotion and progression of HCC^27^. In our study, to further validate the carcinogenenic function of Gankyrin in HCC, we reconfirmed the abnormal expression of Gankyrin in HCC tissues and cell lines compared with the normal controls. Therefore, inhibition of Gankyrin via different methods might be an attractive and promising strategy for controlling HCC development. Hence, additional investigations are needed to explore the mechanism of the aberrant upregulation of Gankyrin and expand its therapeutic potential in HCC.

Notably, accumulating evidence shows that lncRNAs modulate gene expression as epigenetic modifiers^28,29^. Thus, we hypothesized there might exist lncRNAs which might play as important epigenetic modifiers of Gankyrin and then performed microarray analysis to systematically investigate the aberrant expression of lncRNAs in the progression of HCC. Followed hierarchical clustering analysis and VENNY analysis identified 6 lncRNAs which the greatest comparative differential expression. Further expression verification based on small sample and Pearson correlation analysis based on Gankyrin expression validated a brand-new LincRNA ENST00000413791.1, and we named it as Linc-GALH (Gankyrin Associated LincRNA in Hepatocellular carcinoma). Then, we extended the study to explore the expression of Linc-GALH in expanded samples which further validate the abnormal expression spectrum in HCC. Next, in a cohort of 108 HCC patients, according to the expression of Linc-GALH in HCC tissues, we analyzed the association between Linc-GALH expression and the clinical characteristics of HCC patients, and the results indicated that high expression of Linc-GALH may promote the metastasis of HCC. The survival analysis revealed that Linc-GALH upregulation was correlated with shorter overall and recurrence-free survival time.

Portal vein invasion and metastasis is a fatal step in HCC metastasis^30^. In our study, the HCC patients with PVTT had much higher Linc-GALH expression compared with those without PVTT. Therefore, Linc-GALH may be a novel risk biomarker for judging HCC metastasis. The followed functional experiments validated that Linc-GALH play an important role in HCC metastasis. We have also analyzed the expression of EMT related genes, which play a vital role in HCC metastasis, and the results demonstrated that Linc-GALH could promote the EMT transform in HCC. In general, our results implied that Linc-GALH may be closely associated with HCC metastasis.

DNA methylation is an epigenetic mechanism that regulates gene transcription by adding a methyl group to the CpG dinucleotide cytosine^31^. In particular, promoter methylation of tumor-suppressor genes results in transcriptional silencing and loss of gene function, subsequently driving cancer development^32^. High levels of methylation of Gankyrin were found in metastatic gastric cancer tissues, which were significantly higher than those in non-metastatic gastric cancer patients and controls^11^. Since we have revealed that the expression of Linc-GALH was accordance with Gankyrin in HCC patients, we assumed that DNA methylation might participate in the expression of Gankyrin in HCC patients and Linc-GALH might be involved in this regulation. Followed experiments demonstrated that Linc-GALH could regulate Gankyrin expression through modulating DNA methylation and DNMTs might take part in this mechanism. Numerous studies have proved that DNA methyltransferases (DNMTs, including DNMT1, DNMT3A and DNMT3B) are the main factors involving in the regulation of oncogenes and anti-oncogenes in various tumors including HCC, and DNMT1 is the most abundant type of DNMTs which catalyzes DNA methylation^33–35^. Therefore, we designed to explore whether all the DNMTs or one of them play a part in the regulatory relationship. Interestingly, we found that only DNMT1 participated in this regulation and the further in-depth study revealed that Linc-GALH could accelerate the degradation of DNMT1 through enhancing ubiquitination and then promote the expression of Gankyrin by reducing the methylation state in HCC.

In summary, we revealed that Linc-GALH could regulate the expression of Gankyrin through controlling the methylation status of Gankyrin by adjusting the ubiquitination status of DNMT1 in HCC. Since Linc-GALH was found to affect the development of HCC, we expect that Linc-GALH can serve as a predictive marker for HCC and target for preventive drugs.

## Methods and materials

### Patient samples and cell lines

Data were obtained from 108 paired HCC fresh tissues (including tumors and adjacent normal samples) and 12 normal liver tissues (hepatic hemangioma patients) acquired between August 2012 and September 2013 at The First Affiliated Hospital of Nanjing Medical University (Nanjing, Jiangsu, China). Informed consent for tissue analysis was obtained before surgery. The study was approved by the Institutional Ethics Committee of Nanjing Medical University. All research was performed in compliance with government policies and the Helsinki declaration. All experiments were undertaken with the understanding and written consent of each subject. The Huh7, SNU-423, MHCC-97H, MHCC-97L, SMMC-7721, Hep3B, HepG2 human hepatoma cell lines, and the human normal liver L02 cell line were obtained from KeyGen (Nanjing KeyGen Biotech Co., Ltd., Jiangsu, China). The cells were cultured in Dulbecco’s modified Eagle’s medium (DMEM, Invitrogen Life Technologies, Carlsbad, CA, USA) supplemented with 10% fetal bovine serum (FBS, Gibco, Carlsbad, CA, USA) at 37 °C in humidified air containing 5% carbon dioxide.

### Quantitative real-time PCR

Total RNAs of fresh tissue samples and cells were extracted with TRIzol reagent according to the manufacturer’s instructions (Invitrogen, CA, USA). The qRT-PCR was conducted to evaluate the expression level of related lncRNAs and the mRNAs of all relevant genes. GAPDH was used as the internal control, and all primers used were presented in Supplementary Table 1.

### Ectopic expression and gene silencing

The shRNA sequences targeting Linc-GALH and Gankyrin were cloned into lentivirus vector GV248 (Gene, Shanghai, China), respectively, and the negative control shRNAs without sequence homology to human genes were provided by the same manufacturer. All shRNA sequences are presented in Supplementary Table 2. Knockdown efficiencies are shown in Supplementary Figure 3A, C. For overexpression of Linc-GALH, sequence of Linc-GALH was subcloned into the lentiviral vector GV367 (Gene, Shanghai, China). All vectors were labeled with luciferase. Transfection was conducted according to the manufacturer’s instructions.

### Western blot

To analyze the protein, tissue samples and cultured cells were dissolved using a RIPA buffer(50 mM Tris, 1.0 mM EDTA, 150 mM NaCl, 0.1% Triton X-100, 1% sodium deoxycholate, 1 mM PMSF) (Beyotime, Nantong, China). Consistently, 30 μg of protein was loaded in each lane, fractionated by SDS-PAGE, and transferred onto a PVDF membrane. Then, the membrane was incubated at 4 °C overnight with human specific Gankyrin (Abcam, ab182576), DNMT1 (Abcam, ab13537), DNMT3A (Abcam, ab4897), DNMT3B (Abcam, ab79822), Ubiquitin (Cell Signaling, 3933), phospho-AKT(Thr308)(Cell Signaling, 13038), AKT(Cell Signaling, 4685), E-cadherin (Cell Signaling, 3195), N-cadherin (Abcam, ab18203) Vimentin (Abcam, ab137321), Snail (Abcam, ab53519) and β-actin (Cell Signaling, 4970) antibodies. The results were visualized by a chemiluminescent detection system (Pierce ECL Substrate Western blot detection system, Thermo Scientific, IL, USA).

### Cell migration and invasion assays

For would healing assay, cells were seeded at a density of 4 × 10^4^ cells/cm^2^. On day 3, a straight scratch was made with a 200 ml pipette tip and images of the wound acquired under the microscope with the original magnification of 40×. After 48 h, cells were photographed under the microscope and the remaining scratch area calculated. Cell migration was determined using Transwell chambers (8 μm pore size, Millipore, Darmstadt, Germany). Cells were cultured in the upper chamber with serum-free medium. After 48 h, cells that had migrated or invaded through the membrane were fixed with methanol, stained with crystal violet and counted.

### Lung metastasis model

A total of 32 6-week old male node mice were randomly and blindly divided into four groups, and subjected to anesthesia under ketamine (100 mg/kg, intraperitoneal) and xylazine (20 mg/kg, intraperitoneal). Cells transduced with different lentiviruses were suspended in 200 μl phosphate-buffered saline and filtered through a sterile 70 μm nylon mesh (BD Falcon, Franklin Lakes, NJ, USA) to obtain a single-cell suspension. Next, cells were injected into mice through the tail vein. Mice were killed after 6 weeks and tumor metastasis in the lung monitored with the IVIS Lumina II system (Caliper Life Sciences, Hopkinton, MA, USA). Tissues were examined via hematoxylin and eosin staining to evaluate tumor numbers.

### Immunohistochemical assay

The tissue samples were fixed in 4% paraformaldehyde at 4 ℃ and sectioned into slices. After deparaffinizing and rehydration, the sections were put into a pressure cooker for 5 min to restore the antigen in the nucleus using the citrate method. To reduce the background, H_2_O_2_ was used to suppress the endogenous peroxidase activity. The samples were blocked in normal goat serum with 5% BSA in TBS for 1 h at room temperature. The sections were incubated with primary antibody (1:400 dilutions) overnight at 4 °C and then washed with PBS three times. After incubation with secondary antibodies, the sections were subjected to a DAB reaction. The sections were photographed using a digitalized microscope camera (Nikon, Tokyo, Japan). Using Image-Pro Plus software (v. 5.0), average values of integrated optical density (IOD) was obtained by analyzing five random fields per slide. Every index was detected a minimum of three times.

### Bisulfite sequencing

Genomic DNA was isolated and digested with EcoRV (Takara) at 37 °C for 6 h, boiled for 5 min to stop the reaction, treated with NaOH at the final concentration of 0.3 M and denatured at 37 °C for 15 min. 2x volume of 2% low melting agarose was added to the DNA solution and mixed by pipetting. Agarose beads were formed by mixing 10 µl aliquots of DNA solution with cold mineral oil. Beads were transferred to the tubes containing 1 ml of modifying solution (2.5 M sodium bisulphite [2.5 M sodium metabisulphite and 2 M NaOH] and 1 M Hydroquinon) and incubated at 50 °C for 12 h in the darkness. The resulting beads were washed 3 times in TE buffer (10 mM Tris-HCl [pH 8.0], 1 mM EDTA [pH 8.0]), 2 times in 0.2 M NaOH and 3 more times in TE buffer. Beads were then washed 3 times in double distilled water and used for PCR amplification with the corresponding primers. PCR products were cloned into the pMD18-T vector (Takara) and sequenced.

### Statistical analysis

Statistical tests included *t*-tests, *χ*^2^ tests, and Mann-Whitney *U* tests. Data are presented as means ± s.e.m. Pearson correlation analysis was applied to analyze the relationships between associated factors. Survival curves were assessed by the Kaplan-Meier method, and differences in Linc-GALH expression were examined using log-rank tests. Results with *P* < 0.05 were considered significant. All statistical tests were conducted using SPSS (SPSS, Palo Alto, CA, USA), all statistical data were analyzed and presented using GraphPad Prism 5.0 (GraphPad Software, La Jolla, CA, USA). *P*-values < 0.05 were considered statistically significant.

## Supplementary information

Supplementary Information
