# Supplementary material for: The long non-coding RNA Linc-GALH promotes hepatocellular carcinoma metastasis via epigenetically regulating Gankyrin
Source: Cell Death Dis. 2019 Jan 28;10(2):86. doi: 10.1038/s41419-019-1348-0 (PMC6349924; doi:10.1038/s41419-019-1348-0)
Supplement: Supplementary file 1 — Supplementary Information [file 41419_2019_1348_MOESM1_ESM.docx]

**Supplementary Information for:**

**The long non-coding RNA Linc-GALH promotes** **hepatocellular carcinoma metastasis via epigenetically regulating Gankyrin**

Xiaoliang Xu^2,4,#^, Yun Lou^1,4,#^, Junwei Tang^1,#^, Yue Teng^3^, Zechuan Zhang^1,4^, Yin Yin^4^, Han Zhuo^1^, Zhongming Tan^1,*^

^1^ Key Laboratory of Living Donor Liver Transplantation, Department of Liver Surgery, National Health and Family Planning Commission, The First Affiliated Hospital of Nanjing Medical University, Nanjing, Jiangsu, P.R. China.

^2^ Medical School of Southeast University, Nanjing, Jiangsu, P.R. China.

^3^ Department of Medical Oncology， Jiangsu Cancer Hospital & Jiangsu Institute of Cancer Research & Nanjing Medical University Affiliated Cancer Hospital, Nanjing, 210009, Jiangsu, P.R. China.

^4^ Department of Hepatobiliary Surgery, The Affiliated Drum Tower Hospital of Nanjing University Medical School, Nanjing 210093, Jiangsu Province, P.R. China.

# These authors contributed equally to this work.

* Correspondence author: Dr. Zhongming Tan, Key Laboratory of Living Donor Liver Transplantation, Department of Liver Surgery, National Health and Family Planning Commission, The First Affiliated Hospital of Nanjing Medical University, Nanjing, Jiangsu, P.R. China. E-mail: Seektzm@hotmail.com.

**Contents:**

1. **Supplementary figures**
2. **Supplementary tables**
3. **Supplementary figures:**

**
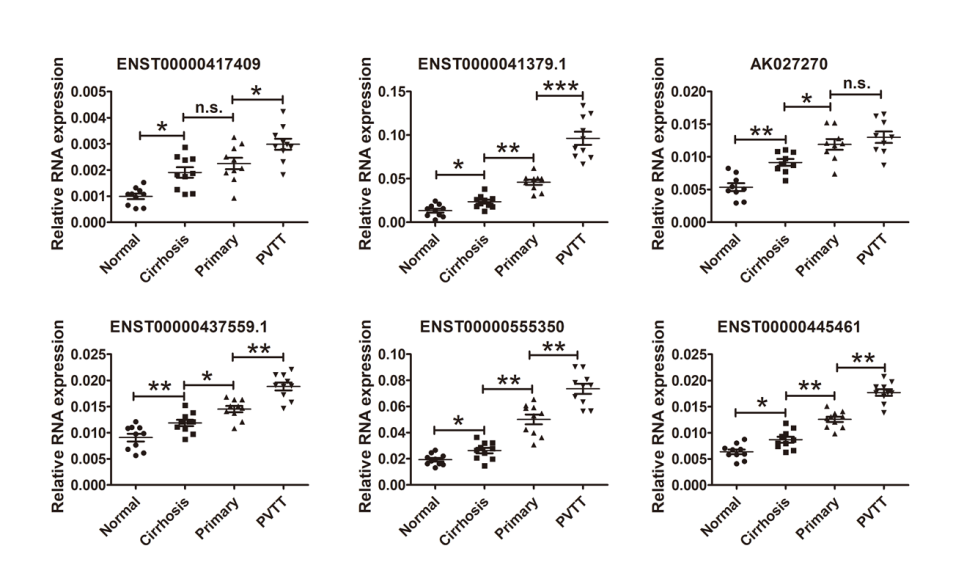
**

**Supplementary Figure 1:** The expression levels of the identified 6 lncRNA was detected in expanded samples (10 normal liver tissues, 10 cirrhosis liver tissue, 10 primary liver cancer tissues and 10 tissues from HCC patients with PVTT). *** *P*<0.001, ***P*< 0.01 and **P*< 0.05. *P*<0.05 was regarded as statistically significant.

**
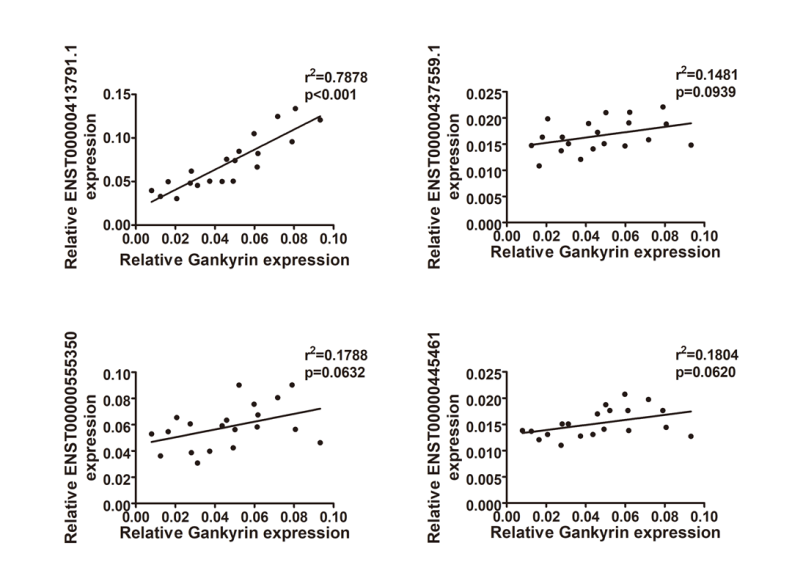
**

**Supplementary Figure 2：**Pearson analysis was conducted to calculate the expression correlation between the expression of Gankyrin and the expression of the lncRNAs detected above in HCC tissues.


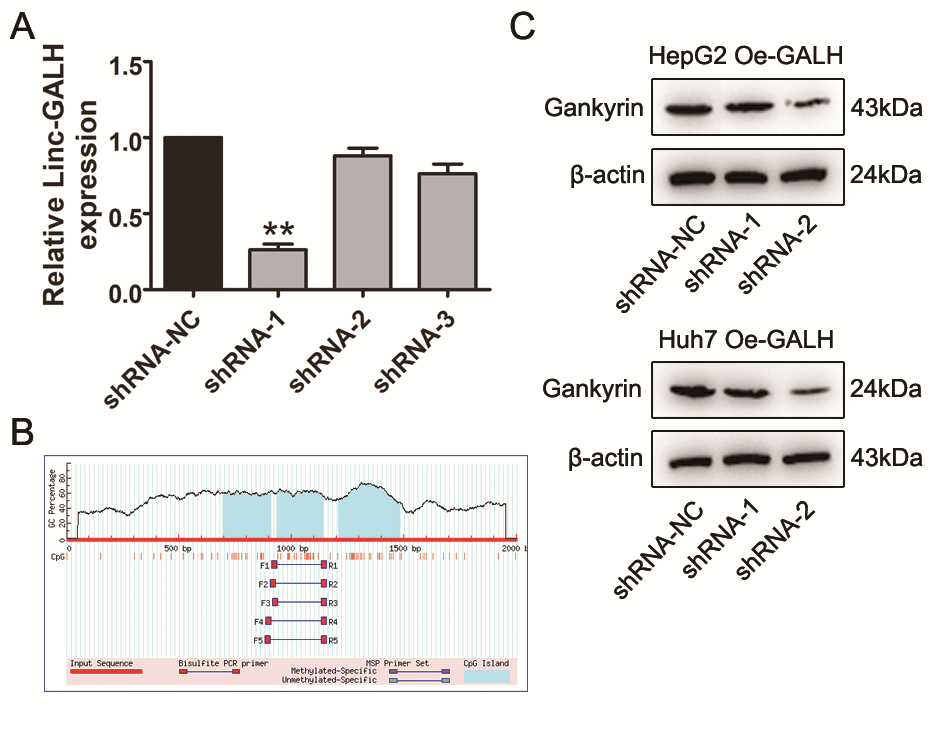


**Supplementary Figure 3:** (A) The knockdown efficiency of Linc-GALH in SMMC-7721 cells was detected by qPCR compared with the control group. (B) Methprimer was used to predict the CpG Island in the promoter region of Gankyrin. ***P*< 0.01. *P*<0.05 was regarded as statistically significant. (C) The transfection efficiency of Gankyrin in Linc-GALH overexpressed HepG2 and Huh7 cells was examined by western blot.

1. **Supplementary tables:**

**Supplementary Table 1. Primers for Quantitative RT- PCR and methylation**

| **Gene name** | **All Patients** | **Sequence** |
| --- | --- | --- |
|  |  |  |
| Gankyrin | Forward Primer | GGGTGTGTGTCTAACCTAATGG |
|  | Reverse Primer | GGCCAGAATACTCTCCTTCAACT |
| Linc-GALH | Forward Primer | AGATTGCGCCAACCCTTACA |
|  | Reverse Primer | ATCCCCGACATTGTGTGACC |
| GAPDH | Forward Primer | GGAGCGAGATCCCTCCAAAAT |
|  | Reverse Primer | GGCTGTTGTCATACTTCTCATGG |
| ENST00000417409 | Forward Primer | CAGCAGCATCTTGTTTGCAG |
|  | Reverse Primer | CTTAACCTCGAAGCCCTCTG |
| AK027270 | Forward Primer | AAAGTAGCCACACAGCACTCAG |
|  | Reverse Primer | AGGGAGATATGGCAAATAGGC |
| ENST00000437559.1 | Forward Primer | TGGTGTAAAGTGGAGGCACA |
|  | Reverse Primer | CACCCGTAGCACAAACTTGTAA |
| ENST00000555350 | Forward Primer | GGTTCACCAACAGAGCAGAAC |
|  | Reverse Primer | GTGGAGTTGGTATCTTAAGTCATTACA |
| ENST00000445461 | Forward Primer | GATCATAGGAGAAAACCATTTCAGA |
|  | Reverse Primer | TCCTCCATTAAGTTTTCTGCATC |
| Gankyrin | Forward Primer | GGGGTTAAGTTTGTGAGGGTAGTA |
| (Bisulfite PCR) | Reverse Primer | CAAACCATTAAATTAAACACACACC |

**Supplementary Table 2. Summary of shRNA Oligos**

| **Name** | **Oligo Sequence** |
| --- | --- |
| Linc-GALH shRNA 1 | GGAGCAATTGACTGAGTAGTCCGAAGACTACTCAGTCAATTGCTCC  CCTCGTTAACTGACTCATCAGGCTTCTGATGAGTCAGTTAACGAGG |
| Linc-GALH shRNA 2 | GGTGTTGTCATAAGAAGAGAGCGAACTCTCTTCTTATGACAACACC  CCACAACAGTATTCTTCTCTCGCTTGAGAGAAGAATACTGTTGTGG |
| Linc-GALH shRNA 3 | GCTGCTGCAAACTAAGGAATGCGAACATTCCTTAGTTTGCAGCAGC  CGACGACGTTTGATTCCTTACGCTTGTAAGGAATCAAACGTCGTCG |
| Gankyrin shRNA 1 | GCATGCTCAGCTGGACATACACGAATGTATGTCCAGCTGAGCATGC  CGTACGAGTCGACCTGTATGTGCTTACATACAGGTCGACTCGTACG |
| Gankyrin shRNA 2 | GCCTGGGTTTAATACTCAAGACGAATCTTGAGTATTAAACCCAGGC  CGGACCCAAATTATGAGTTCTGCTTAGAACTCATAATTTGGGTCCG |

**Supplementary Table 3. The 6** differentially expressed lncRNAs

| **LncRNA** | **Chromosome** | **LncRNA-start** | **LncRNA-end** | **Strand** |
| --- | --- | --- | --- | --- |
| ENST00000417409 | chr1 | 190449509 | 190463427 | + |
| ENST00000437559.1 | Chr6 | 12007670 | 12008856 | + |
| AK027270 | chr2 | 136686257 | 136687847 | - |
| ENST00000413791.1 | Chr2 | 70275819 | 70314553 | - |
| ENST00000555350 | Chr14 | 28103331 | 28105875 | + |
| ENST00000445461 | chr21 | 17566788 | 17979542 | + |
